# Taking the Bite

**Published:** 1906-08

**Authors:** Stewart J. Spence

**Affiliations:** Chattanooga, Tenn.


### TAKING THE BITE.

BY STEWART J. SPENCE, CHATTANOOGA, TENN.

(Continued from last month).

As the path of the condyle sometimes loses its normal inclination (which is about that of an arc of a circle of 1¼ inches radius) and sometimes becomes less inclined, or even straight, especially in old persons, its inclination should be determined and transformed to the articulator. For this purpose only the methods No. 2 and No. 3 can be used. It may be done thus: After taking the bite as far as above described, have patient protrude lower jaw to its utmost, then mark the amount of protrusion by a scratch on wax or plate. Now, if the condyle's path is normally arched there will be a space between the posterior occlusal surfaces; if straight, no space. In the former case, place a knife blade between, draw it forward till tight, mark its position by scratch on wax, and so place models on articulator that when the lower jaws is protruded to the extent marked, the knife blade will go to its place. If this is not done, you may find that your finished denture occludes, in the laberal or masticatory bite, earlier on one side of the mouth than on the other, thus loosing for your patient the great benefit arising from correct simultaneous occlusion.

If Dr. Snow's face-bow is used it should be adjusted to the tempero-maxillary articulation (about half inch in front of the ear-hole) if for articulators having their joints on the same plane as their upper jaw; but if for articulators (like the Kerr) which open at the middle, the face bow should be adjusted to a spot about one and a half inches vertically below said meatus auditorium (ear-hole).

